# Revolutionizing Chronic Kidney Disease Management with Machine Learning and Artificial Intelligence

**DOI:** 10.3390/jcm12083018

**Published:** 2023-04-21

**Authors:** Pajaree Krisanapan, Supawit Tangpanithandee, Charat Thongprayoon, Pattharawin Pattharanitima, Wisit Cheungpasitporn

**Affiliations:** 1 Division of Nephrology and Hypertension, Department of Medicine, Mayo Clinic, Rochester, MN 55905, USA; pajaree_fai@hotmail.com (P.K.); supawit_d@hotmail.com (S.T.); charat.thongprayoon@gmail.com (C.T.); 2 Division of Nephrology, Department of Internal Medicine, Faculty of Medicine, Thammasat University, Pathum Thani 12120, Thailand; pattharawin@hotmail.com; 3 Division of Nephrology, Department of Internal Medicine, Thammasat University Hospital, Pathum Thani 12120, Thailand; 4 Chakri Naruebodindra Medical Institute, Faculty of Medicine Ramathibodi Hospital, Mahidol University, Samut Prakan 10540, Thailand

Chronic kidney disease (CKD) poses a significant public health challenge, affecting approximately 11% to 13% of the global population [1]. This accounts for over 800 million people worldwide [2]. Over the past two decades, the burden of CKD has grown at a faster rate than that of other noncommunicable diseases [3,4]. This is likely due to a considerable rise in cardiovascular events, kidney failure requiring renal replacement therapy, poor quality of life, and mortality [1]. CKD is projected to become the fifth-leading cause of death worldwide by 2040, with an estimated 5 to 10 million deaths annually [1,2]. Survivors of CKD often experience a range of systemic complications, including cardiovascular disease, hypertension, anemia, mineral bone disorder, volume overload, electrolyte and acid-base abnormalities, malnutrition, sexual dysfunction, and pruritus, which can adversely affect their quality of life [1,2].

The top three leading causes of CKD are diabetes mellitus (DM), hypertension, and primary glomerulonephritis, which account for 70–90% of all cases worldwide [1,2]. Additionally, numerous factors influence the progression of CKD, including modifiable risk factors such as diabetes, hypertension, proteinuria, body mass index, smoking, and nephrotoxic medications, as well as non-modifiable factors such as age, gender, ethnicity, family history of kidney disease, and low socioeconomic status [1,2]. Early detection of patients at risk is crucial to delaying kidney disease progression. This can be achieved through the measurement of eGFR and the urinary albumin-to-creatinine ratio (ACR), as well as interventions related to nutrition, lifestyle, and medications to control blood pressure and glucose levels and reduce albuminuria [1,2].

Despite the existence of evidence-based guidelines for managing CKD and the demonstrated ability of current treatment care models to delay CKD progression and improve patient outcomes, the global nephrology community recognizes that these approaches are inadequate for addressing the growing burden of CKD [1,2]. Given the multitude of risk factors and diagnostic tests involved, accurately diagnosing, predicting the prognosis of, and optimally managing CKD can be challenging for clinicians [5]. In response to this complex problem, artificial intelligence (AI) has been introduced as a potential solution [6,7,8,9].

AI is the ability of a human-made machine to display complex decision-making or data analysis compared to human intelligence [6,10,11,12,13,14]. Machine learning is a subset of AI that involves teaching machines to recognize patterns and make predictions from data without explicit programming [15,16,17,18,19].

As shown in Figure 1, the two primary categories of machine learning are supervised and unsupervised learning [20,21,22]. Supervised learning uses labeled data to train machine learning models to recognize patterns and make predictions. Examples of supervised learning in the context of CKD include the diagnosis of CKD solely from kidney ultrasound or fundus imaging, predicting kidney progression, mortality, and hemoglobin level in hemodialysis patients receiving erythropoietin stimulating agents (ESAs), as well as identifying optimal treatment for patients [20,21,22,23].

On the other hand, unsupervised learning uses unlabeled data to identify patterns or clusters without prior knowledge of the outcome. Examples of unsupervised learning in CKD include clustering patients based on similar clinical and demographic characteristics [7,9] or discovering unknown biomarkers or subtypes of the disease [8].

The use of machine learning and AI has the potential to revolutionize the management of CKD [20,21,22]. These technologies can help with early detection and diagnosis by analyzing large datasets of patient health records, identifying patterns, and predicting those at risk of developing CKD [20,24]. Machine learning algorithms can also aid in detecting kidney damage through the analysis of medical images and personalize treatment by identifying the most effective treatment for each patient based on their clinical and demographic characteristics [20,21,22]. Predictive analytics can help identify patients at risk of developing complications, and remote monitoring can allow clinicians to track patient health in real-time. Additionally, machine learning and AI can help identify new treatment and prevention strategies and provide personalized treatment plans for CKD treatment.

While several pharmacological treatments, including sodium–glucose cotransporter-2 (SGLT2) inhibitors, renin–angiotensin system (RAS) inhibitors, glucagon-like peptide-1 (GLP-1) agonists, nonsteroidal mineralocorticoid receptor antagonists (MRAs), and combination therapies, have demonstrated potential in attenuating the progression of CKD and improving cardiovascular outcomes [4], the efficacy of these treatments can differ depending on the characteristics of individual patients. Thus, utilizing machine learning in future research could help tailor treatments for CKD patients and determine which patients may derive the most benefit from each intervention. Machine learning algorithms can assess vast amounts of patient data to identify correlations and associations between patient features and treatment responses, resulting in personalized treatment recommendations that could enhance outcomes for CKD patients.

Recently, a language model, ChatGPT (https://chat.openai.com/), has been developed and will require additional study to validate if ChatGPT can accurately provide a variety of resources and information related to CKD that could potentially enhance patient care in the future. It can potentially offer educational resources to both patients and healthcare providers, including details on CKD causes, symptoms, and treatment options. The language model can also facilitate communication between patients and healthcare providers, ensuring that patients receive the information they require to manage their condition effectively. Furthermore, ChatGPT can improve access to information on CKD, including the latest research and treatment options. Despite its limitations in the ability to diagnose or treat CKD, ChatGPT’s resources and support could potentially improve patient care for this chronic condition in the future.

Overall, machine learning has the potential to improve our understanding of CKD and provide personalized treatment plans for individual patients.

## Figures and Tables

**Figure 1:**
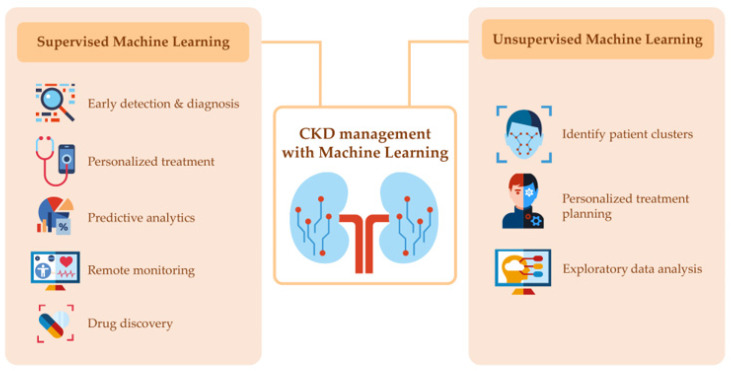
Representation of the two categories of machine learning in chronic kidney disease management.
